# Special Issue: Nutraceutical Approaches to Cardiovascular and Metabolic Diseases: Evidence and Opportunities

**DOI:** 10.3390/nu14245399

**Published:** 2022-12-19

**Authors:** Paolo Magni, Andrea Baragetti, Andrea Poli

**Affiliations:** 1 Department of Pharmacological and Biomolecular Sciences, Università degli Studi di Milano, 20133 Milan, Italy; 2 IRCCS Multimedica Hospital, 20099 Milan, Italy; 3 Nutrition Foundation of Italy, 20124 Milan, Italy

The effective prevention and treatment of cardiovascular and metabolic diseases is a major task for health systems since these pathological conditions are still major causes of mortality, morbidity, and disability worldwide. To pursue this aim, strategies should be adopted to take advantage of lifestyle changes, including, when appropriate, pharmacological approaches and considering the nutraceutical option, which, according to an increasing research and practical interest, appears to be an additional and effective asset in this biomedical field. This Special Issue of *Nutrients* has thus been devoted to the discussion of the current experimental and clinical evidence regarding the efficacy and the safety of nutraceutical products for managing cardiometabolic diseases, also taking into consideration critical issues such as the quality of nutraceutical products, the related regulatory aspects, and the quality of evidence required to inform guidelines.

Among papers reporting experimental studies, Ullah et al. approached the potential nutraceutical properties of a hydroethanolic extract of *Prunus domestica* L. The authors were able to identify the extract’s active components and explore its anti-inflammatory activity and ability to inhibit digestive enzymes involved in some features of the metabolic syndrome [1]. Moreover, Atchan Nwakiban et al. assessed the potential anti-obesity nutraceutical activities of 11 Cameroonian medicinal spice extracts in a human-cell-based model of human adipocytes [2].

Shifting to clinical studies, Domínguez-López et al. highlighted that the level of urinary tartaric acid, a biomarker of wine intake, correlated with lower total and low-density lipoprotein (LDL) cholesterol in postmenopausal women, suggesting that wine consumption may have a positive effect on the cardiovascular risk profile, thus exhibiting some nutraceutical properties [3]. Another paper discusses the beneficial effects of the dietary use of nuts in terms of cardiometabolic diseases, leading Ros et al. to suggest that nuts can be considered natural pleiotropic nutraceuticals [4]. The role of carotenoids, another family of compounds with nutraceutical properties, is then reviewed by Lem et al. in the context of managing diabetic retinopathy [5]. Moreover, a real-world study by Tragni et al. showed that a very-low-calorie ketogenic diet results in a significant reduction in cardio-metabolic risk, in addition to weight loss, in women with overweight/obesity [6], while a study by Baragetti et al. identified a significant relationship between gut microbiota functional dysbiosis and the individual diet in subjects in primary cardiovascular disease (CVD) prevention [7]. Another aspect of the relationship between diet and CVD was addressed by Mattavelli et al., who discussed the complex physiopathological relationship between specific dietary fats, inflammation, and cardiovascular disease and the contrasting data from the literature regarding observational studies and interventional trials [8]. The most common and robust nutraceutical approaches used in clinical practice for cardiovascular and metabolic disease prevention and treatment are presented in a paper by Banach and Penson [9]. Some of these approaches are then discussed in greater detail in other papers in this issue. Cicolari et al. show data on the interactions of oxysterols with atherosclerosis biomarkers in subjects with moderate hypercholesterolemia in relationship with a nutraceutical combination including a probiotic and red yeast rice extract [10]. Phytosterols are a family of nutraceutical compounds with well-established functional properties: their use and effectiveness in cholesterol control and cardiovascular disease management are discussed in detail by Poli et al. [11]. This Special Issue then closes with a critical evaluation of the inclusion (or not) criteria of nutraceutical options in the most relevant guidelines for obesity, diabetes mellitus, and dyslipidemias, highlighting the strengths and limitations of the available evidence (Casula et al.) [12].

This Special Issue of *Nutrients* aims to implement a qualified and open evidence-based discussion on the use of nutraceutical products for cardiometabolic health, thus providing an up-to-date set of information useful for readers involved in experimental/clinical research as well as clinical practice in this biomedical area.

## References

[1] Ullah H., Sommella E., Santarcangelo C., D’Avino D., Rossi A., Dacrema M., Minno A.D., Di Matteo G., Mannina L., Campiglia P. (2022). Hydroethanolic Extract of *Prunus domestica* L.: Metabolite Profiling and In Vitro Modulation of Molecular Mechanisms Associated to Cardiometabolic Diseases. Nutrients.

[2] Atchan Nwakiban A.P., Passarelli A., Da Dalt L., Olivieri C., Demirci T.N., Piazza S., Sangiovanni E., Carpentier-Maguire E., Martinelli G., Shivashankara S.T. (2021). Cameroonian Spice Extracts Modulate Molecular Mechanisms Relevant to Cardiometabolic Diseases in SW 872 Human Liposarcoma Cells. Nutrients.

[3] Domínguez-López I., Parilli-Moser I., Arancibia-Riveros C., Tresserra-Rimbau A., Martínez-González M.A., Ortega-Azorín C., Salas-Salvadó J., Castañer O., Lapetra J., Arós F. (2021). Urinary Tartaric Acid, a Biomarker of Wine Intake, Correlates with Lower Total and LDL Cholesterol. Nutrients.

[4] Ros E., Singh A., O’Keefe J.H. (2021). Nuts: Natural Pleiotropic Nutraceuticals. Nutrients.

[5] Lem D.W., Gierhart D.L., Davey P.G. (2021). A Systematic Review of Carotenoids in the Management of Diabetic Retinopathy. Nutrients.

[6] Tragni E., Vigna L., Ruscica M., Macchi C., Casula M., Santelia A., Catapano A.L., Magni P. (2021). Reduction of Cardio-Metabolic Risk and Body Weight through a Multiphasic Very-Low Calorie Ketogenic Diet Program in Women with Overweight/Obesity: A Study in a Real-World Setting. Nutrients.

[7] Baragetti A., Severgnini M., Olmastroni E., Dioguardi C.C., Mattavelli E., Angius A., Rotta L., Cibella J., Caredda G., Consolandi C. (2021). Gut Microbiota Functional Dysbiosis Relates to Individual Diet in Subclinical Carotid Atherosclerosis. Nutrients.

[8] Mattavelli E., Catapano A.L., Baragetti A. (2021). Molecular Immune-Inflammatory Connections between Dietary Fats and Atherosclerotic Cardiovascular Disease: Which Translation into Clinics?. Nutrients.

[9] Penson P.E., Banach M. (2021). Nutraceuticals for the Control of Dyslipidaemias in Clinical Practice. Nutrients.

[10] Cicolari S., Pavanello C., Olmastroni E., Puppo M.D., Bertolotti M., Mombelli G., Catapano A.L., Calabresi L., Magni P. (2021). Interactions of Oxysterols with Atherosclerosis Biomarkers in Subjects with Moderate Hypercholesterolemia and Effects of a Nutraceutical Combination. Nutrients.

[11] Poli A., Marangoni F., Corsini A., Manzato E., Marrocco W., Martini D., Medea G., Visioli F. (2021). Phytosterols, Cholesterol Control, and Cardiovascular Disease. Nutrients.

[12] Casula M., Catapano A.L., Magni P. (2022). Nutraceuticals for Dyslipidaemia and Glucometabolic Diseases: What the Guidelines Tell Us (and Do Not Tell, Yet). Nutrients.

